# Factors affecting effective community participation in maternal and newborn health programme planning, implementation and quality of care interventions

**DOI:** 10.1186/s12884-017-1443-0

**Published:** 2017-08-31

**Authors:** Lisa Howard-Grabman, Andrea Solnes Miltenburg, Cicely Marston, Anayda Portela

**Affiliations:** 1 Training Resources Group, Inc., 4301 Wilson Boulevard, Suite 400, Arlington, VA 22203 USA; 2 Institute of Health and Society, Department of Community Medicine and Global Health, Faculty of Medicine, University of Oslo, P.O. Box 1072 Blindern, 0316 Oslo, Norway; 3 Faculty of Public Health and Policy, London School of Hygiene & Tropical Medicine, 15-17 Tavistock Place, London, WC1H 9SH UK; 4 World Health Organization Department of Maternal, Newborn, Child and Adolescent Health, 20 Avenue Appia, 1211 Geneva, Switzerland

**Keywords:** Community participation, Maternal and newborn health, Quality improvement, Health programme planning and implementation

## Abstract

**Background:**

Community participation in in health programme planning, implementation and quality improvement was recently recommended in guidelines to improve use of skilled care during pregnancy, childbirth and the postnatal period for women and newborns. How to implement community participation effectively remains unclear. In this article we explore different factors.

**Methods:**

We conducted a secondary analysis, using the Supporting the Use of Research Evidence framework, of effectiveness studies identified through systematic literature reviews of two community participation interventions; quality improvement of maternity care services; and maternal and newborn health programme planning and implementation.

**Results:**

Community participation ranged from outreach educational activities to communities being full partners in decision-making. In general, implementation considerations were underreported. Key facilitators of community participation included supportive policy and funding environments where communities see women’s health as a collective responsibility; linkages with a functioning health system e.g. via stakeholder committees; intercultural sensitivity; and a focus on interventions to strengthen community capacity to support health. Levels of participation and participatory approaches often changed over the life of programmes as community and health services capacity to interact developed.

**Conclusion:**

Implementation requires careful consideration of the context: previous experience with participation, who will be involved, gender norms, and the timeframe for implementation. Relevant stakeholders must be actively involved, particularly those often excluded from decision making. Current limited evidence suggests that the vision of community participation as a process and the presence of a focus to strengthen community capacity to participate and to improve health may be a key factor for long term success;

## Background

Community participation in health is: ‘a process whereby people, both individually and in groups, exercise their right to play an active and direct role in the development of appropriate health services, in ensuring the conditions for sustained better health and in supporting the empowerment of community to help development’ p.10 [1]. Involving communities in assessing their own needs and in developing strategies to meet those needs can increase intervention ownership and sustainability, while responsiveness to community needs in planning and implementation of health programmes can help improve health equity, service delivery, and uptake of care [2–4]. Various reviews and World Health Organization (WHO) Guidelines have highlighted the importance of community participation for improved health [5–9].

The WHO commissioned systematic reviews of health promotion interventions involving community participation. We performed a secondary analysis on two of them here [10]: 1) quality improvement of maternity care services where community members participate in processes to review the quality of health services either as informants or as partners with health providers in planning and implementation to improve quality; and 2) maternal and newborn health programme planning and implementation, where community members are involved in planning, designing, implementing and monitoring strategies and interventions. Based on these reviews, community participation in quality improvement and in health programme planning and implementation is now recommended by WHO to improve use of skilled care during pregnancy, childbirth and the postnatal period for women and newborns, increase the timely use of facility care for obstetric and newborn complications and improve maternal and newborn health [10]. In addition to the available evidence on the impact of participation, it is also important to understand which factors influence implementation of community participation interventions for maternal and newborn health. This article addresses this question, exploring stakeholder perspectives and experiences of the two community participation interventions, and identifying barriers and facilitators to successful implementation.

## Methods

We analysed the studies included in systematic reviews of published and unpublished grey literature used to inform WHO health promotion guidelines for maternal and newborn health [10]. The methods for the review are described in the WHO document.

The systematic reviews included articles published between 2000 and 2012 initially identified from a systematic mapping of maternal health research in low- and middle-income countries [11]. Studies included RCTs as well as any other study design that included at least one data collection point prior to the intervention and one during or after the intervention. Studies reporting qualitative data were included. The systematic reviews themselves are not the topic of this paper.

We extracted data from 16 studies that could shed light on factors influencing implementation using an adapted ‘SURE (Supporting the Use of Research Evidence) framework’ [12]. The framework includes a comprehensive list of barriers and facilitators to implementing health systems interventions including stakeholder knowledge and attitudes, health service delivery factors, and social and political considerations. Starting from the categories within the broad SURE framework the authors identified further, specific themes of interest from the primary empirical data presented in the included articles and from the author discussion and conclusions from those articles.

## Results

### Description of included studies

Table 1 shows characteristics of included studies. 16 papers reported on 13 separate programmes: seven in Asia (India *N* = 1; Bangladesh *N* = 1; Pakistan *N* = 2; Nepal *N* = 1; China *N* = 1, Indonesia *N* = 1), three in East Africa (Tanzania *N* = 1; Uganda *N* = 1; Kenya *N* = 1) and three in Latin America (Peru *N* = 2; Honduras *N* = 1). Implementation approaches for community participation varied. They included forming stakeholder committees [13–21], mobilizing communities to take action [14, 22–25], community based monitoring of health outcomes or services [17–19, 25, 26], community outreach activities to increase awareness of health issues [13, 16, 27] and facilitating stakeholder dialogues [19, 28]. Many of the studies were complex, multiple intervention programmes that combined community participation with health system strengthening and some also combined multiple approaches to participation. There was no consistent definition of ‘community’ and some studies did not define ‘community’ at all.

**Table 1:** Characteristics of studies and description of interventions

No	Study	Setting	CP^a^	Time frame	Approach	Level	General description of intervention or aim of the study
1	Purdin S, et al. (2009). Reducing maternal mortality among Afghan refugees in Pakistan	Pakistan: Hangu district of Khyber Pakhtunkhwa Province (rural refugee settlements)	P&I	1980–2007`	Community outreach and stakeholder committee	Outreach	Provision of reproductive health services for Afghan refugees through establishment of Basic Health Units and Basic Emergency Obstetric Care facilities. Camp-based health committees included community representatives who attended bi-monthly meetings with health staff to discuss project activities and provide feedback to providers on services provided. The Basic Health Unit staff trained Community Health Workers and committee members including men on safe motherhood and reproductive health topics to educate others in the refugee community.
2	Ahluwalia I, et al. (2003). An evaluation of a community-based approach to safe motherhood in northwestern Tanzania (See also Ahluwalia, 2003)	Tanzania: Kwimba Missungwi districts (rural)	P&I	1998–2000	Community mobilizing	Outreach, Consult, Involve	As part of a Community Based Reproductive Health Project (CBRHP) strengthening of community level services was done through a special activity called the Community Capacity Building and Empowerment Project. The project aimed for local problem solving through 1) training, technical assistance, and support for (village health workers) VHWs who provided educational house visits on topics such as recognition of danger signs and birth preparedness; [2] developing community-based plans for transportation to health facilities and [3] increasing participation by community members in planning and decision-making through community meetings, aiming to identify and solve local health problems.
3	Ahluwalia I, et al. (2010). Sustainability of community-capacity to promote safer motherhood in northwestern Tanzania: what remains? (See also Ahluwalia, 2010)	Tanzania: Kwimba Missungwi districts (rural)	P&I	2006	Community mobilizing	Outreach, Consult, Involve	This study reports on a follow-up study of Ahluwalia (2003) with the aim to examine the remains of the CBRHP as described above. Activities continued from 2001 to 2006 without project support. A post project assessment was conducted with focus on the CBRHP components, including community supported transport systems; village health workers; and changes in selected maternal health service use indicators at the district level.
4	Bhutta Z, et al. (2011)	Pakistan: 2 towns in Sindh with 1400 villages (rural)	P&I	2006–2008	Stakeholder committee and Community mobilizing	Outreach	Community-based intervention package principally delivered through training of Lady Health Worker (LHW) and *Dais* (traditional health workers) and promotion of liaison between them together with facilitation of the creation of voluntary community health committees (CHC). In addition to advocacy work with community elders and local political leaders, the CHCs were encouraged to organize an emergency transport fund and the use of vehicles using local resources. The CHCs facilitated the LHWs in accessing women and in conducting group education sessions in the intervention villages.
5	Paxman J, et al. (2005). The India Local Initiatives Program: A Model for Expanding Reproductive and Child Health Services	India: 4 northern states in Kolkata, the hills of the Himalayas, Punjab plains, and mountains of Himachal Pradesh (urban & rural)	P&I	1999–2003	Stakeholder committee	Involve	NGOs help to organize or strengthen a reproductive and child health committee composed of influential community members. The committees recruit, train and oversee the work of community health volunteers (CHVs), raise money for health activities and support, and enlist support of local government, social and religious leaders. CHVs provided health information to households and kept track of their health status, provided some basic health services including some family planning methods, organized educational activities and referred clients to additional services outside their communities. CHVs tracked health status making use of a pictorial map to facilitate use by people with limited literacy skills, which helped project staff to monitor performance.
6	Kaufman J, et al. (2012). Improving reproductive health in rural China through participatory planning	China: Dafang and Zhenning counties in Guizhou Province, Luoping County in Yunnan Province (rural)	P&I	2002–2006	Community outreach	Involve	The Gender and Health Equity Network (GHEN) project aimed to improve health further for poor rural women by increasing women’ s participation in planning and resource allocation through capacity building through training of township women’s representatives and local officials in gender and health. Women’s health promotion groups and demonstration households were established. Demonstration households collected information on local health service needs and shared this with the health promotion team who communicated to higher-level health authorities. Women and their families were taught how to prevent and treat common health problems and were motivated to use services. Health education activities were organized at least once per month. County and township supervision meetings were held once every two months to provide direction, identify and solve problems.
7	Harkins T, et al. (2008). The health benefits of social mobilization: experiences with community-based Integrated Management of Childhood Illness in Chao, Peru and San Luis, Honduras	Peru: Chao district (peri-urban) and Honduras: San Luis district (rural)	P&I	2004–2005	Stakeholder committee, community outreach	Involve	Multiple government agencies, private sector and non-governmental organizations along with representatives of community-based organizations established or strengthened existing committees that were tasked by the project with disseminating key Integrated Management of Childhood Illness health messages to their various constituencies through their networks with the aim of involving families and communities in maternal and child health approaches. Members of the committee were trained and they in turn capacitated community members. The committees could be creative about how they disseminated the messages. The committee was responsible for organizing the training and events and the supporting logistics for their activities.
8	Sood S, et al. (2004). Measuring the effects of the SIAGA behavior change campaign in Indonesia with population-based survey results	Indonesia, West-Java	P&I	1999–2004	Community mobilizing	Involve	Social mobilization campaign consisted of a mass media component that targeted husbands (Suami SIAGA), birth attendants (Bidan SIAGA), and communities (Warga SIAGA) and villages (Desa SIAGA) with radio and television spots and shows that modeled the desired attitudes and behaviors of “alert” husbands, midwives and communities that support the health of their mothers and babies. There was also a community participation component for the “alert village” that built on a traditional concept of the value of community help. This component aimed at motivating people to establish life-saving systems in their villages (transport, emergency funds, blood)
9	Mathur, et al. (2004). Youth Reproductive Health in Nepal – is participation the answer? (See also Malhotra, 2005)	Nepal: Nawalparasi and Kawasoti Districts (rural Terai) & two urban suburbs of Kathmandu	P&I	1998–2004	Stakeholder committee	Shared Leadership	A youth centered participation project was initiated through a formative research process, which included a needs assessment on how issues of youth reproductive health were relevant in the communities of interest. The project staff facilitated an action planning process through which results of the needs assessment were shared with community members. The project established two community-based advisory groups, the Adolescent Coordination Team (ACT) and the Project Advisory Committee (PAC) consisting of adults. This was followed by formation of separate task forces consisting of youth representatives to develop interventions and an intervention plan. The task forces then came together to integrate their plans after seeking advice from resource people in the community. This was followed by implementation of the interventions. This study documents the process and results of the project.
10	Malhotra, et al. (2005). Nepal: The Distributional Impact of Participatory Approaches on Reproductive Health for Disadvantaged Youth (See also Mathur, 2004)	Nepal: Nawalparasi and Kawasoti Districts (rural Terai) & two urban suburbs of Kathmandu	P&I	1998–2004	Implementation planning through youth involvement taskforces	Shared Leadership	This study reports on the impact of participatory approaches in improving youth reproductive health as reported by Mathur (2004). The authors examine whether the participatory or the non-participatory intervention approach is more successful in reducing the gaps between the disadvantaged and the advantaged in access to youth reproductive health services and in outcomes.
11	Kaseje D, et al. (2010). Evidence-based dialogue with communities for district health systems’ performance improvement	Kenya: 6 districts in Nyanza Province: Nyando, Siaya, Kisumu, Rachuonyo, Suba, Bondo (urban & rural)	P&I, QI	2005–2007	Stakeholder committees, facilitation of dialogue, community based monitoring	Collaborate	An evidence-based dialogue model was introduced to community members, district health management teams, and service providers through a series of three, three-day workshops. The intervention package included the development of committees at the village, community and health facility levels; identify, train and deploy Community Health Extension Workers (CHEWs) as facilitators of dialogue at the community level, supporters of CHWs, and maintainers of a community-based information system; identify and train CHWs to support households in health improvement activities, maintain village register and facilitate dialogue at household level; establishment of village registers of all households; improvement and timeliness of analysis, dissemination and utilization of health management information system data; analyze suggestions collected from suggestion boxes on a monthly basis; and, hold dialogue sessions based on data from the community and health facilities every month at household and community levels and every four months at health facility and sub-district levels. In dialogue sessions, data were displayed, discussed and consensus was built on what was acceptable and what needed to be improved.
12	Bjorkman M, and Svensson J (2009). Power to the People: Evidence from a randomized field experiment on community-based monitoring in Uganda	Uganda: 50 communities from 9 districts in all four regions of Uganda (rural)	P&I, QI	2004–2006	Community-based monitoring	Collaborate (most villages), Shared Leadership (some villages)	With the aim to strengthen providers’ accountability to citizen-clients an NGO-facilitated approach was implemented. First community members were presented with baseline information (a “report card”) which was a summary of information gathered from both community members and service providers as well as data collected from service registers to reflect the status of health service delivery relative to other providers and the government standards. During community meetings community members developed a shared view on how to improve service delivery and monitor the providers. A facility meeting was held with health facility staff to present the results of the household survey and contrast the results to the results of the information provided by services providers. An interface meeting between community representatives elected at the earlier community meeting and health service providers discussed proposed suggestions for improvement and came to agreement on an action plan and a plan for how the community would monitor progress. After six months, health facility staff and community members jointly assessed and analyzed progress.
13	Sinha D (2008). Empowering communities to make pregnancy safer: an intervention in rural Andrha Pradesh.	India: Mominpet in Rangareddy District in Andhra Pradesh (rural)	QI	2004–2006	Community mobilizing and community based monitoring	Shared Leadership (some villages)	Community organizers raised awareness of village councils and youth organizations about the powerful role they could play in ensuring that public health facilities provide the services they are required to deliver and instructed them on how to use a monitoring tool to compare actual performance with expected service delivery. Village councils then held regular monthly meetings to which they invited representatives of local organizations, youth groups, schools, mother’s committees, and community level health workers. Participants in the meetings reviewed service performance, health data and service utilization statistics, identified problems and worked to solve them. When solutions did not work, they initiated action with higher authorities. Meetings were also held at the lower levels. Youth leaders, initially young men but later joined by young women, organized meetings in the villages to raise awareness of young people to hold providers accountable for good service. Eventually, the young people formed a “Youth Committee for Right to Health” that met monthly.
14	Gabrysch S, et al. (2009). Cultural adaptation of birthing services in rural Ayacucho, Peru.	Peru: All 17 villages in the Santillana district, Ayacucho region (rural)	QI	1997–2001	Facilitation of dialogue	Consult, Collaborate	Program cycle approach to engaging pregnant women and health providers in the development of maternity services that met both service provider and community expectations for quality care. Phase 1: detailed formative research by project team to understand perceptions and practices related to reproductive health and health services. Phase 2: 3 facilitated meetings of pregnant women, TBAs, CHWs and health providers to design a birthing service that was ` to all parties. Phase 3: Implementation of newly designed birthing service through capacity building workshops for health providers and TBAs to teach each other; sharing of evidence-based practices; informing of the population about the new service. Phase 4: Project evaluation followed by minor adaptation to the model. Phase 5: Routine monitoring and assessment of sustainability until 2007.
15	Barbey A, et al. (2001). Dinajpur SafeMother Initiative Final Evaluation Report (see also Hossain & Ross, 2006)	Bangladesh: Dinajpur & Panchagarh in northwestern Bangladesh (rural)	QI	1998–2001	Stakeholder committees and community based monitoring	Involve	This study reports on an evaluation of the Dinajpur Safe motherhood Initiative (DSI) to examine and validate the achievements, and explain the attribution of the specific project interventions. The DSI had the primary aim of testing the impact of a defined package of interventions. Facility interventions included facility upgrades to provide basic Emergency Obstetric Care (EmOC) and improvements of quality of care through the creation of Stakeholder Committees, with representation of health providers and 11 community members (leaders, active TBAs, CBOs) to build rapport between the community and the health care system and through the enhancement of health service provider capacity. The stakeholder committee met regularly and monitored service cleanliness and client perceptions of services, as well as reviewed maternal death or near-miss cases. Community interventions included: birth planning education through home visits and group discussions at clinic and village meetings by SBAs, fieldworkers and village doctors who were trained to disseminate BP messages that were also incorporated into a variety of visual aids; the establishment of Community Support System (CmSS) including emergency funds for EmOC, emergency transportation for referral to another health facility, identification of volunteers to accompany women to facilities or to provide financial support and a list of volunteers who are available to donate blood in case of emergency. DSI specifically aimed to ensure quality services for all women subjected to violence, particular during pregnancy.
16	Hossain & Ross (2006). The effect of addressing demand as well as supply of emergency obstetric care in Dinajpur, Bangladesh (see, also Barbey et al., 2001)	Bangladesh: Dinajpur & Panchagarh in northwestern Bangladesh (rural)	QI	1998–2001	Stakeholder committees and community based monitoring	Involve	This study reports on the impact of the Dinjpur Safe motherhood Initiative (DSI) as reported by Barbey (2001) on utilization of EmOC services.

^a^CP – Community Participation in P&I (Programme planning and implementation), QI (Quality Improvement) and/or MDSR (Maternal Death Surveillance and Response)

Community participation did not always fit neatly into one category, ranging from communities being the recipients of health messages to high level engagement where community members and groups played active roles in decision-making, planning and implementation [29]. In six programmes, community members participated at different levels at different points during the intervention [17, 18, 22, 23, 25, 26, 28]. Communities were involved in designing programmes from the beginning in only two cases [19–21]; in four programmes, communities provided input on interventions [25–28]; and in seven, programme teams designed the programme and chose the interventions. The communities were then asked to adapt and implement them [13–18, 22–24]. Women participated at lower levels than men in many of the studies, [13, 14, 17, 18], however, one study focused exclusively on women’s participation [27].

### Implementation barriers and facilitators

Tables 2 and 3 present barriers and facilitators to successful implementation across the two distinct interventions.

**Table 2:** Facilitators of implementation cited in studies included in the systematic review for each research question

Facilitators of implementation	Community participation in quality improvement	Community participation in MNH programme planning & implementation
Enabling/supportive environment		
A supportive political environment with supportive policies makes it easier to implement programmes.	18, 19	28
Community awareness of and interest in MNH are high.		
• When mortality is high, it is more likely that community members will see the problem and perceive the need for change. • Use mass media campaigns (radio/TV) and other outreach methods to increase awareness of the issue.	18, 19	17, 25
Reinforce or nurture cultural norm of collective responsibility for better maternal & newborn health.		23, 24
Build on and/or develop more cohesive populations with tighter social networks. Rural programme sites had an easier time implementing than those in urban sites in part due to more cohesive populations, tighter social networks.		16, 21, 22
Community capacity		
*Community leadership*		
Having strong and stable community leadership facilitates implementation.	18, 19, 27	16, 18, 23, 24, 27
Improve community leadership, ownership & governance of programme	18, 20, 26, 27	16, 18, 20
Provide women/young people with opportunities for leadership, forum for participation	18, 20, 26	16, 18, 20, 21, 22, 28
Increase focus and attention to health in local council meetings	26, 27	25, 27
COMMUNITY PARTICIPATION & GOVERNANCE		
Ensure representation of the voices and perspectives of different groups	20, 26, 27	17, 20
Increase participation of marginalized, disadvantaged, less powerful groups	26, 27, 29	21,22, 27
Increase women’s participation in decision-making		23, 24, 28
Work with existing structures when they are functional or have flexibility to form new structures/mechanisms when they don’t exist or are dysfunctional (need to understand their purpose, roles and responsibilities). Establish and/or strengthen committees or other planning & coordination structures.	18, 19, 20, 26, 27	14, 15, 16, 17, 18, 21, 22, 23, 24, 25, 27, 28
COLLABORATION & PARTNERSHIP		
Establish and/or strengthen multi-organization partnership including public sector/local government at multiple levels.	18, 19	16, 17, 23, 24, 25, 28
Improve community - health services interaction/relations.	18, 19, 20, 29	15, 18, 20, 21, 22, 27
Increase awareness and support of community health workers.		14, 15, 23, 24
Strengthen social networks for information exchange/support.		21, 22, 25
• Violence against women advocacy support network established, action taken to address this issue	18, 19	18, 19
COMMUNITY MANAGEMENT CAPACITY		
Strengthen community ability to use data for decision-making, monitoring, accountability & advocacy.		
• Communities, households, services with more complete data; using data	20, 26	14, 20
• Use of data for decision-making, advocacy	20, 26, 27	
• Improved community monitoring and accountability of health services	18, 19, 26, 27	21, 22, 27
Strengthen community ability to leverage and manage resources.		
• Transparency in decision-making and management of resources	18, 19, 20	18, 20, 23, 24
• Community capacity to leverage and manage resources	18, 20	16, 20, 23, 24, 25
Strengthen community ability to plan; development of written action plan, “community contract” that guided implementation.	18, 19, 20, 27	20, 21, 22, 23, 24, 27
Strengthen community ability to problem-solve.	18, 19	18
*Community capacity: health related technical knowledge, skills & abilities*		
Train village health workers/community volunteers to be able to provide health education and services.	19, 20, 26	14, 16, 17, 23, 24
Develop blood donor lists to identify potential donors, if needed.	18, 19	18, 19, 25
Improve knowledge of danger signs.	18, 19	17,18, 19
Health system		
Sufficient number of trained staff in health facilities		14, 15
Improve quality of care/upgrade services	[aim of all studies for this intervention]	14, 15, 18
Availability of accurate data on health situation, health services	19, 20, 27	16, 20, 27
Leadership at district and health facility levels	18, 19	
Community & health system interaction		
Community health workers play a vital role linking communities and health services	18, 19, 20, 26, 29	14, 15, 16, 20, 23, 24
NGOs can facilitate the process, provide technical support to communities to help them develop capacity to plan and implement. Existing relationships of NGOs with communities and health services facilitate implementation. NGOs can support inter-cultural interaction.	18, 19, 27, 29	14, 16, 17, 18, 23, 24, 27
Bring communities and health service providers together to participate in joint assessment and dialogue before planning.	20, 29	20, 28
Use key questions to drive planning process dialogue.	20, 29	20
Schedule regular meetings (monthly, bimonthly, quarterly) to monitor, adjust strategies, problem-solve.	18, 19, 20, 26, 27	
Intercultural sensitivity /competence		
Acknowledge and build on existing traditional/local beliefs and practices.	29	25
Develop/use culturally appropriate materials in local languages that are suitable for the range of literacy/numeracy skills in the programme context.	18, 19, 27, 29	15, 16, 18, 25, 27
Understand social networks and focus on changing social norms.	26	21, 22, 25
Maintain a gender rights focus and consider gender roles.	18, 19	16, 18, 25, 28
Other programme conditions		
Use participatory methodology and techniques		21, 22, 25
Use a synergistic package of complementary interventions		18
Provide funding support for a longer period of time (this study was funded for 4 years)		28
Train programme facilitators (in MNH topics, data interpretation, dissemination, conflict resolution, management)		14, 15, 17, 18, 23, 24, 27

*Note: see numbered list of references at the end of the article to interpret the numbers presented in the columns below. This is a descriptive, qualitative analysis based on what the reviewed studies reported. The number of studies reporting each facilitating factor is not intended to be an indicator of the level of importance of the factor.*

**Table 3:** Implementation barriers and challenges cited in studies included in the systematic review for each research question

Implementation barriers & challenges	Community participation in quality improvement	Community participation in MNH programme planning & implementation
Not-so-enabling environment		
Need more supportive maternal health policies		1, 2
Low status of women, gender inequity	18, 19, 26	14, 28
Discrimination against indigenous people, ethnic groups, poor people	29	
Conflict, insecurity and violence against women	18, 19, 29	14
Politicians do not collaborate when they see no benefit for themselves		16
Urban environment highly politicized		16
Urban setting negatively affects time available to participate, especially for men; recruitment and retention of community health volunteers is also more challenging.		16, 21, 22
Community capacity		
*Community leadership*		
Changes in leadership		15
Community leadership doesn’t prioritize maternal health or health more generally.	19	
COMMUNITY GOVERNANCE & MANAGEMENT		
Community capacity to plan and work together is limited. Takes time to develop.	20	16, 20, 21, 22, 23, 24, 28
Trust issues exist among different groups.	18, 19	16, 18, 22
• Lack of transparency in management of community funds.	18, 19	18
Ineffective structures		
• Existing structures are dysfunctional	27	27
• At sub-district level, organizational structures are less defined and many different local groups exist. (Dinajpur Safe Motherhood Initiative chose to develop a Community Support System structure to address this challenge.)		18, 19
Health system		
Managing resources & resource constraints		
• Human resource constraints of public health system		15
• Health services supervision system weak, irregular	27	27
• Services lack “modern equipment and advanced technology”	18, 19	18, 21, 22
Health facility data inconsistent and incomplete – difficult to plan effectively and difficult to assess attribution of programme outcomes; limited capacity for data management	18, 20, 29	18, 20
Service provider attitudes are resistance to change	29	21, 22
Wider health system issues such as ineffective referral system (outside of local control)	29	
Community -health system interaction		
MANAGEMENT OF RESOURCES & RESOURCE CONSTRAINTS		
• Limited access to facilities (distance, difficult terrain)	20	17, 20
• Lack of funds (for transport)	20	20
• Lack of financial and technical resources (MOH, community)	20	20, 23, 24
• Rotation of health personnel doesn’t allow time to develop trusting relationships with community	29	
• Expectations of community health workers are unrealistic; too many tasks		15
Poor communication	20	20
Need to improve linking/interface of communities with services	18, 19	18, 23, 24
Intercultural sensitivity/competence		
Cultural traditions of women delivering and residing in other homes outside of study area for postnatal period affects birth preparedness plans and postnatal follow-up care.		15
Reluctance of families to travel long distances for neonatal care (cultural practice and security issues underlie this reluctance)		15
Increasing empowerment of youth led to conflict at times		21, 22
Reaching and including people with low literacy and numeracy skills	29	17
May not be reaching the poorest and most vulnerable with the strategies used, strategies may not be effective for these groups	18, 19	18
General programme design/implementation challenges		
Proxy indicators have some limitations (e.g., utilization of EmOC for “met need”)	18, 19	18
Expansion and scaling up	20, 26, 29	
Low coverage and high complexity of the intervention		15
Volunteers taking on too many tasks		15

*Note: see numbered list of references at the end of this article to interpret the numbers presented in the columns below. This is a descriptive, qualitative analysis based on what the reviewed studies reported. The number of studies reporting each barrier or challenge is not intended to be an indicator of the level of importance of the factor.*

We identified five categories of implementation barriers and facilitators reported by the studies: 1) the extent to which there was an enabling and supportive environment or not; 2) the nature of community capacity; 3) health system factors; 4) features of the interface between community and health services; and, 5) intercultural competence and sensitivity of the programmes.

The findings across the two interventions were very similar so in this analysis we discuss them together. However, Tables 2 and 3 provide the reader with specific details about which factors were reported in each study.

#### Enabling and not-so-enabling environments

The Millennium Development Goals triggered supportive maternal and newborn health policies and political commitment at the highest levels of government in many countries which changed the overall context for these programmes. Community participation interventions in Bangladesh, India, Peru, China, and Indonesia were implemented in the context of new government schemes and approaches to upgrade services and make them more accessible and affordable [16–18, 24, 25, 27]. For example, through the National Rural Health Mission, the Indian government introduced subsidies and incentives to make services more accessible and affordable for pregnant women and their families [25]. In China, a rural health insurance scheme was introduced which allowed local officials to decide on which services would be covered. This, in turn, created opportunities for programme participants to advocate for more accessible services for women [27]. At the district and village levels, NGOs involved in establishing health committees with the India Local Initiatives Programme characterised the urban environment as highly politicized with disputes that interrupted progress. The study also reported that politicians did not collaborate with the programme when they saw no benefit for themselves [15].

Cultural norms of collective responsibility helped communities to plan and work together to address barriers to accessing quality care. In Indonesia, the SIAGA social mobilization project intentionally built on the traditional value of collective help (*gotong royong*) as the foundation for their “alert community” campaign. This aimed at motivating people to establish life-saving systems in their villages (transport, emergency funds, blood) [24]. In Tanzania, one study showed how community members initially perceived women’s health as the responsibility of individuals and were not inclined to work together to address barriers to service use [22, 23]. As the programme evolved, however, the study authors report that community awareness grew: both about the nature of the challenges and how they could help by working together. Over time, the members of the community began to value collective responsibility and action [22, 23]. The communities where this shift in norms occurred were also reportedly more likely to sustain their efforts to improve health and maintain mechanisms such as transport systems compared with those that remained focused on individual responsibility [23].

#### Community capacity

Studies reported many facilitating and inhibiting factors related to community capacity development, both generally in terms of community leadership, governance and management, and more specifically in relation to health knowledge, skills and abilities. Many programmes worked with committees and stakeholder groups that helped facilitate the participation process. In Bangladesh and Kenya, lack of transparency in decision-making and management of resources led to the committees dissolving and compromised the trust necessary for villagers to work together successfully [17–19]. Bhutta, et al. (2011) observed that leadership transitions were a challenge to implementation; trust and relationship building had to begin again with each new leader [14]. Eight of the studies noted the value of multiple organizations at multiple levels working in partnership, recognizing that improving maternal and child health would require participation and support of many stakeholders [15–18, 22–24, 27]. The majority of studies reviewed that worked with committees reported that committees were most successful when their purpose and individual roles and responsibilities within the committees were clear [13]. Strong and stable community leadership was highlighted as a key facilitator to effective implementation in six studies [15, 17, 18, 22, 23, 26]. While many of the studies opted to involve leaders and influential people in quality improvement stakeholder committees and groups, some authors emphasized the importance of ensuring representation for those who often did not have a voice in community planning and implementation. They underscored the importance of providing opportunities for women and young people to develop and exercise leadership skills and have a forum for their participation on issues that clearly and directly affect and interest them [16, 17, 19, 25, 26]. In general, studies reported value in having regular meetings to monitor progress, adapt strategies as necessary and solve problems [17–19, 25, 26].

A key factor influencing implementation in most studies included whether interventions helped communities address the issues that affected them. Some programmes were designed with this aim in mind; for example, the youth participation programme in Nepal and the Gender and Health Equity Network in China helped groups of socially marginalised people learn how to influence decision-making on health policy making and practice [20, 21, 27]. In Uganda, the programme “encouraged communities to be more involved with the state of health service provision and strengthened their capacity to hold their local health providers to account for performance” [26]. In other studies this type of process occurred not by design, but serendipity: the community mobilization study in India, for instance, relied on the programme’s community organizers to act as intermediaries between communities and health services as part of the intervention which in turn increased community willingness and ability to hold health workers accountable for services [25]. Studies that did not include elements of community capacity development ran into implementation challenges. For example, several studies describe how community, facility and government stakeholders needed time to develop ways of planning and working together which did not always fit programme timelines [15, 19–23, 27]. Sometimes communities had limited understanding of how to interpret and manage health data, which hindered effectiveness of community based monitoring [17–19].

#### Health system factors

Limitations within health systems were highlighted in many studies. For instance, five studies highlighted the importance to health facilities of having accurate data on population health, health services and case studies of maternal deaths and “near-misses” to improve quality and planning within services, as well as to share with the broader community to raise awareness about health priorities and to monitor progress over time [15, 17–19, 25, 26]. Yet, incomplete and inconsistent data at health facilities made it difficult to plan effectively, and also made it hard for programmes to assess the effects of changes they had made [17, 19, 28]. Resource constraints also presented challenges to effective implementation [14, 14, 17, 18, 20, 21]. Two studies reported that having trained staff in health facilities and upgrading the quality of care helped facilitate work with communities [13, 14]. A persistent challenge in many health programmes that was also evident in the studies reviewed here was a weak, irregular and ineffective supervision system for healthcare staff [26]. Barbey (2001) indicates that health system leadership at the district and facility levels is key to quality improvement efforts [17].

#### Interface between community and health services

Communities and health services may face challenges in coming together to plan and implement programmes. Some communities had limited access to facilities because of distance, difficult terrain and lack of funds for transport, while health service providers may face problems trying to reach communities and supervise community health workers [16, 18, 19]. Poor communication and lack of financial and technical resources on both sides (public health sector and community) can limit communities’ and service providers’ ability to meet, which can limit the effectiveness of such partnerships in improving healthcare [19, 22, 23].

Several studies reported that joint assessments between healthcare providers and members of the community helped improve quality by providing valuable information to feed into priority setting and identify opportunities and challenges [19, 27, 28]. Community health workers, volunteers, and NGOs play important roles in linking communities with health systems by facilitating dialogue, providing health education and services through community outreach, collecting health and community data, and by drawing on existing relationships which help them understand the local context and priorities [13–15, 17–19, 22, 23, 25, 28]. Sometimes, however, volunteers were expected to take on too many tasks and thus could not complete all of them well [14]. Barbey et al. (2001) advise that facilitators be well-trained in facilitation, coaching and training skills [17].

#### Intercultural competence and sensitivity of the programmes

Culturally-appropriate materials in local languages are needed that are suitable for a range of literacy and numeracy skills for programmes where community members participate in analysis of health data as a basis for decision-making and action [14, 15, 17, 18, 24, 28]. Programmes in Indonesia, India and Nepal highlighted the importance of programme personnel understanding and working to mobilize social networks in culturally-sensitive ways to bring about changes in social norms [20, 21, 24, 25]. A gender roles analysis study in China advocated a gender rights focus as a way to help raise community awareness about inequities in women’s access to services and other opportunities, making the case for women’s participation in bottom up planning processes in resource-poor settings where women’s status is low to better inform decision-makers about women’s needs and views [27].

Women’s low status appears to have influenced how community priorities were set, how decisions were made at the household level, and also influenced women’s level of participation. Gender inequity manifested in different ways in different places. For instance, in Peru, Quechua women were discriminated against and treated poorly by health services staff. Ongoing local conflict also affected their sense of security and limited access to health facilities [16]. Similarly, in India husbands were reluctant to participate in maternal health interventions, describing maternal health as a “women’s issue.” Study authors reported, “It was clear that efforts to make husbands more supportive questioned deep-rooted norms and beliefs, and met with considerable resistance; consequently, husbands were slow to change their views.” [27]. The studies in Bangladesh showed how women suffered from violence in multiple settings: at home, in communities and in health services [17, 18].

### Benefits and harms

Reported benefits of community involvement in monitoring health data and quality included increased accountability of the health system to the community. Studies also reported reduced absenteeism [25, 26], reduced drug stock-outs [19, 26], reduced waiting time [26], better examination procedures [26], improved facility infrastructure and equipment [19, 25, 27] and reduced use of untrained providers [25] as well as generally improved quality of care [17, 18]. Other cited benefits included policies and actions that reflected and addressed women’s needs [27].

At community level reported benefits included improved abilities of individuals, groups and communities in governance of programmes [15, 17, 19, 25, 26], management, planning and using data for group decision-making [13, 19–23, 25, 26], obtaining and managing resources [15, 17, 19–24], facilitating group processes to include new voices [20–23, 25–28], monitoring and evaluation, conflict management, and problem-solving [17, 18, 20, 21, 24–26]. In addition, participation interventions helped community members improve their knowledge and skills about maternal and newborn health, which enabled them to carry out specific health related tasks or functions [13, 14, 17, 18, 22, 23]. Two studies suggested that community capacity development can also help sustain improvements in health [20, 23].

Most studies did not report on specific harms. In a study from Peru, the authors noted that it was important to attend to both the personal needs of community members as well as ensure adequate medical quality [28]. In addition, increased empowerment of young people in Nepal sometimes led to conflict when it challenged existing social norms [20, 21].

### Stakeholder perspectives and experiences

The quality improvement studies provided very limited information on stakeholder perspectives. Stakeholder dialogue in developing culturally-acceptable childbirth services in Peru was reported to have helped create mutual understanding between communities and service providers and the new services developed as a result were hailed as a success locally [28]. A study in India [25] using community mobilization and monitoring noted: “Community leaders reported that as a result of direct interaction with government officials on problems faced by health providers and the women themselves, there was more openness among officials to resolving issues and a greater willingness to accept feedback from the community” p14 [25]. Women in the same study said there was more community support for pregnancy-related care. Interventions at family level found approaching mothers-in-law helpful, as they were more receptive to community organizers than husbands [25].

Studies on community participation in planning and implementation of health programmes reported positive experiences of programme participants, although they reported few details. Stakeholder committees in Bangladesh were reported to be generally acceptable, with the strongest committees recognizing they could apply their strengthened problem-solving skills to other issues beyond health, and saying that they appreciated programme staff assistance in how they motivated committees to come up with their own solutions [18]. Members of committees in India and Kenya were reported to appreciate having a better understanding of their own roles and responsibilities, for instance in the decision making process for resource allocation and financial management [15, 19]. In Nepal, where young people participated to try to improve reproductive health, ‘community members and, in particular, young people, felt a strong enough sense of ownership over the project to demand accountability from the implementation team. By the end of the project, young people were leading many of the intervention activities, with the implementation team acting only as facilitators’ [20, 21]. Study authors reported that community outreach through dissemination of health messages by health promotion groups or committees was widely accepted in Peru and China [18, 27]. Health providers in Peru reportedly said that families became more knowledgeable about maternal and child health danger signs, and of how to care for children with diarrhoea [16]. Four studies [14, 24–26] did not report any stakeholder perspectives or experiences, including whether or not the intervention was acceptable to them.

## Discussion

### Linking implementation factors to health outcomes

The designs of the original studies, along with the complexity of the integrated multi-component interventions and the different approaches to community participation, make it difficult to link specific implementation strategies to specific outcomes [7, 8]. The studies themselves attribute the following positive outcomes to participation strategies: increased community awareness of danger signs and complications [15, 16, 24]; an increase in appropriate care-seeking [12, 14–23, 25, 26, 30]; improved transport to services, either through financial support being made available after community meetings, or because of increased awareness through educational materials, or broader programme activities [15, 16, 20–24]; and creating a process for community members to use health data to identify and address barriers to survival [15–19, 23, 25].

Rifkin argues that approaching community participation as a process rather than an intervention influences how the effect of community participation should be evaluated [7]. Process evaluations were not usually documented in the studies included here, a finding which is in line with previous reviews [8, 9]. For studies where communities played more active roles, particularly in planning and implementing health programmes [19–21, 25–27], rather than more passive roles as recipients of community outreach [13, 14, 16], development of community capacity to address programme challenges and barriers and increase programme ownership was a key factor, whether or not this was an intended programme goal. Strengthened abilities of community members and groups to plan and implement programme-related activities acquired through experience and training can be applied beyond the programme, enhancing community participation in broader civil society in the immediate and longer term [22, 23, 25, 26]. However, the Nepal youth study suggests that strengthening a community’s capacity to work together effectively without paying careful attention to developing specific health-related knowledge and skills may not result in the desired improvement of specific health outcomes, at least in the short term (presumably in a context in which community level health related knowledge and skills are not well developed) [26, 27]. Strengthened capacity can lead to shifts in the balance of power through partnerships or coalitions between different groups (for example through stakeholder committees) or increased skills, education and confidence of members who become empowered to tackle their own problems (for example through community-based monitoring and increased accountability) [5, 31–34].

### Limitations

Our findings are limited by the following: a relatively small number of studies met the inclusion criteria for the original WHO reviews of effectiveness; there is little detail in these studies about stakeholder perspectives and the context and conditions in which programmes were implemented. Most studies did not report on their definition of ‘community’ and it is unclear whether they had operational definitions or were working with implicit understandings such as a rough geographical definition. The way community is defined has programmatic implications in terms of organization, leadership, representation, governance and decision-making processes, particularly when programme implementers choose to work with existing structures and organizations and so clearly influences the process of community participation. Lack of clear definitions of who comprises ‘community’ suggests a lack of focus in the implementation, which could be problematic – for instance if the intervention amplifies already-heard voices at the expense of marginalised groups.

Across studies there was limited discussion about why the programmes chose the approaches they implemented and the extent to which these approaches seemed to work, or needed to be adjusted during the programme. Authors provided very little information on the process of participation, what motivated different community members to participate, and how their participation contributed to successful outcomes. They also did not discuss certain key details that could inform implementation of programmes in the future, for instance, which theoretical or pedagogical approach(es) they used, the particular roles that community and programme team members played in the learning process or how these roles may or may not have changed over time as community capacity grew and environmental conditions changed. Most authors did not report on how programme team members developed their own capacity to design, facilitate and support these processes and how relationships and personalities influenced effectiveness during implementation.

There is an urgent need for better qualitative data to be collected in future studies to ensure that dynamics and processes are captured to inform future participation programs [4, 7]. Documenting, evaluating and reporting on highly complex and dynamic community participation approaches using conventional evaluation methods and designs with the rigor required to establish a strong evidence base may be difficult for implementers who may lack relevant resources and expertise. Conventional quantitative and qualitative evaluation methods supplemented by systems thinking [35, 36], complexity aware monitoring [37], realist evaluation and other methods [38] may provide greater insight into how these processes work in different contexts and conditions, but such evaluation methods require adequate resources and staff to be done thoroughly.

## Conclusion

While the conclusions from the secondary analysis presented here are necessarily tentative, a key element of successful health programme interventions through community participation appears to be the extent to which community, facility and government stakeholders develop their capacity to work effectively together to design, manage, and monitor health programmes as well as their health-related knowledge and skills. We identified the following factors to consider when supporting community participation programmes:

- *Enabling or not-so-enabling environment* – the extent to which political will, community awareness and sentiment, policies and available resources are supportive of maternal and newborn health and community participation;
- *Community leadership and governance* characterized by stability and strength of local leadership, the extent to which marginalized voices are represented in decision-making and whether and how to work with existing structures;
- *Community management capacity* to leverage and manage resources, use data for decision-making and for planning, monitoring, and accountability;
- *Community and health system capacity to interact* including the roles and relationships that community health workers, NGOs and others can play to link communities and health systems, and the use of regularly scheduled effective processes that use key questions to drive constructive dialogue; and,
- *Intercultural sensitivity/competence* that acknowledges, respects and builds on existing local beliefs and practices, considers gender rights and roles, understands social networks and norms, uses local languages and materials accessible to the range of literacy and numeracy skills within the programme context.

While some facilitating and inhibiting factors identified in this secondary analysis may be beyond the control or influence of a particular programme, it may be helpful for programme designers and implementers to be aware of them and the possible implications they may have for implementation and results. For example, working in a setting in which there is a very low level of trust among members of the community is likely to require a great deal more time and effort spent on building trust before meaningful engagement in joint programme planning can take place.

Better understanding of how best to support community participation processes to improve maternal and newborn health is essential, particularly methods of investigating adequately the effects of different programmes in what are inevitably complex and dynamic social settings.

## Abbreviations

ANC: Antenatal care
MDSR: Maternal death surveillance and response
MNH: Maternal and newborn health
SURE: Supporting the Use of Research Evidence
WHO: World Health Organization
